# Genetic transformation of *LoHDZ2* and analysis of its function to enhance stress resistance in *Larix* *olgensis*

**DOI:** 10.1038/s41598-022-17191-2

**Published:** 2022-07-27

**Authors:** Peiqi An, Ruofan Qin, Qingrong Zhao, Xuefeng Li, Chen Wang, Qing Cao, Hanguo Zhang, Lei Zhang

**Affiliations:** 1 State Key Laboratory of Tree Genetics and Breeding, Northeast Forestry University, Harbin, 150040 China; 2 Liaoning Forest Inventory and Planning Institute, Shenyang, 110122 China

**Keywords:** Genetics, Plant sciences

## Abstract

To study the function of *LoHDZ2* in larch, we first constructed a VB191103-*LoHDZ2*::GUS overexpression vector. Through Agrobacterium-mediated infection, the expression vector was transferred into a larch embryogenic cell line. A stable resistant cell line was subsequently screened, and mature embryos were induced to grow until they developed into seedlings. Antagonistic cell lines were identified at both the DNA and RNA levels. The transgenic cell lines were then subjected to GUS staining, and transgenic cell lines were ultimately identified and obtained. These transgenic cell lines were sequenced to identify differentially expressed genes, and a cluster analysis was performed. The resistant cell lines were cultured under stress conditions involving 20% PEG_6000_ and 200 mM NaCl proliferation media (1/10-BM). After the stress treatment, the contents of peroxidase (POD), malondialdehyde (MDA) and superoxide dismutase (SOD) in both wild-type and transgenic cell lines were measured. The results are summarized below: (1) When the specific fragment of the target gene in the genome of the resistant cell line was amplified. At the RNA level, the expression of the fragment in four resistant lines increased. In addition, GUS staining showed a blue reaction, indicating that *LoHDZ2* was successfully integrated into the larch embryonic cell lines. (2) To verify the accuracy and reliability of the transcriptome data, 10 differentially expressed genes (5 upregulated and 5 down regulated genes) were subjected to qRT-PCR verification. The results showed that the expression trend of the 10 differentially expressed genes was the same as that revealed by RNA-Seq, indicating that the transcriptome data were reliable. (3) The transcriptome sequencing showed that 176 genes were upregulated and that 140 genes were down regulated. Through GO enrichment analysis and KEGG metabolic pathway analysis, the screened differentially expressed genes were related to biological processes such as larch metabolism and response to stimuli, indicating that these genes may be closely involved in the regulation of the larch response to external stimuli, including heat stress, drought stress, metal ion stress and bacterial infection, and may participate in the growth process. (4) After 20% PEG_6000_ treatment, the POD enzyme activity of the transgenic cell line was greater than that of the wild-type; this activity could effectively remove the amount of peroxide produced. The MDA content of the transgenic cell lines was lower than that of the wild-type cell lines, and the accumulation degree of harmful substances was low, indicating that the degree of oxidative damage of the transgenic cell lines was lower than that of the wild-type cell lines. The SOD content of the transgenic cell lines was lower than that of the wild-type cell lines, indicating that the drought resistance of the transgenic cell lines was enhanced. After 200 mM NaCl treatment, although the increase in SOD content was not obvious, the same trend was detected, indicating that the resistance of the transgenic cell lines was indeed stronger than that of the wild-type cell lines. According to the results of previous experiments, after this gene was overexpressed in tobacco, the transformed plants showed obvious dwarfing, which may indicate that the stress resistance of the plant was enhanced. In conclusion, a transgenic larch cell line was successfully obtained, and transgenic larch seedlings were successfully induced. *LoHDZ2* may participate in the response of plants to the external environment, and may participate in the growth and development of *Larix* *olgensis* by affecting plant metabolic pathways.

## Introduction

*Larix* *olgensis* is an important coniferous tree species in China. Its trunk is straight, and its wood is hard, of good quality and durable^1^. In this study, through the exploration of the internal molecular mechanism of *Larix* *olgensis*, growth stress resistance-related genes of *Larix* *olgensis* were identified, and fast-growing, stress-resistant *Larix* *olgensis* was developed by genetic improvement.

Homeodomain leucine Zippers (HD-Zips) are kinds of plant-specific transcription factors belong to the homeobox protein family^2^. Based on characteristics such as sequence conservation, gene structure and physiological function, HD-Zip transcription factors can be divided into four subclasses: HD-Zip I, HD-Zip II, HD-Zip III and HD-Zip IV subclasses^3^. Due to the differences in genetic sequence and protein structure of members of the different subfamilies, HD-Zip transcription factors participate in different plant development processes and regulate different metabolic processes.

The stress response mechanisms of HD-Zip family have been widely studied in many plants. Due to the different subfamily structures of HD-Zip family, its functions are different. According to studies, the gene expression of HD-Zip I subfamily and HD-Zip II subfamily is induced by drought, high salt, ABA and chilling injury, and participates in hormone signal pathway. It regulates plant cell expansion, division and differentiation by interacting with hormone pathway genes and downstream genes, so as to improve plant stress tolerance^4^. HD-Zip I and II as important regulatory subfamily members in response to abiotic stress.

HD-Zip I family has been identified to play an important role in drought and salt stress. Eucalyptus *EcHB1* overexpression strain reduced transpiration rate, reduced water loss of trees and improved plant drought tolerance due to reduced leaf area and no change in stomatal density^5^. In transgenic rice, overexpression of *HDG11* can increase ABA content and enhance stomatal closure by inducing the expression of *OsNCED3*, the key gene of ABA biosynthesis^6^. *HDG11* overexpression leads to ABA hypersensitivity, induces stomatal closure and targets auxin biosynthesis gene YUCC6 and ABA response genes *ABI3* and *ABI5*, indicating that *HDG11* enhances drought and salt stress tolerance through auxin and ABA mediated Chinese Kale^7^. *HDG11* transgenic pepper and cotton poplar have high levels of proline, soluble sugar, antioxidant enzymes (SOD) and cat during high concentration NaCl stress treatment, which reduces the oxidative damage of plants and is conducive to tolerance to salt stress by regulating osmotic homeostasis^8^.

HD-Zip I protein was also involved in regulating fruit ripening. By silencing tomato *LeHB-1* gene through VIGS, the expression of *LeACO1* decreased significantly, and the fruit ripening was inhibited; HD-Zip II proteins are also involved in the regulation of flowering stage. The sunflower HD-Zip II transcription factor *HAHB10* can induce plant flowering^9^. HD-Zip III and HD-Zip IV subfamily members are mainly involved in regulating plant growth and development. HD-Zip III subfamily members are mainly involved in regulating plant apical meristem, vascular bundle and lateral organ development in paraxial region^10^. HD-Zip IV genes are specifically expressed in plant epidermal cells and mainly regulate epidermal cell differentiation, root growth, anthocyanin accumulation and hairy body formation^11^.

Based on previous laboratory-based research results of HD-Zip family genes, the *LoHD-Zip* family genes were analyzed by bioinformatics and qRT-PCR. According to the test results, the genes with large differential expression were selected as the main follow-up research object. To further study the function of these genes, we cloned *LoHDZ2* and overexpressed it in larch calli by the Agrobacterium-mediated method to obtain transgenic larch. The changes in physiological and biochemical indices of transgenic larch under drought and salt stress were then evaluated via transcriptome sequencing, and the function of *LoHDZ2* was preliminarily studied.

## Materials and methods

### Plant materials

Embryogenic cell lines of *Larix* *olgensis* preserved in our laboratory were used as research objects, and fresh embryogenic calli cultured for approximately 14 days were used as plant materials. The wild-type *larix* *olgensis* cell lines used in the experiment were induced in the early stage of the laboratory and stored in the State Key Laboratory of Forest Tree Genetics and Breeding(Northeast Forestry University).The basic medium used was 1/10-BM proliferation medium, and dark culture was carried out at room temperature at 25 °C.

According to the *LoHDZ2* sequence of *Larix* *olgensis* (the gene number in the NCBI database is MW206675). *LoHDZ2* was cloned and its expression vector was constructed, named as VB191103-*LoHDZ2*::GUS*.* Amplification fragments for specific primers were designed, and a VB191103-*LoHDZ2*::GUS plant overexpression vector was constructed. The construct was successfully transformed into competent *Agrobacterium* *tumefaciens* cells for plant genetic transformation^12^.

### Genetic transformation of larch

Fresh embryogenic callus lines were selected from 1/10-BM callus proliferation media for genetic introduction of VB191103-*LoHDZ2*::GUS. The genetic transformation of larch was carried out by Agrobacterium-mediated infection. The calli of larch were infected with infection solution with an OD600 of 0.5 for 20 min and cocultured on media for 2 days. After coculture, the calli were cleaned with sterilized water three times and then transferred to culture media consisting of 500 mg/L Cef for strict sterilization two times (each time for 5 min). After cleaning, the calli were placed on sterile filter paper to absorb excess water. Then they were transferred to screening media consisting of 4 mg/L Hyg to screen for resistant larch calli. After three resistance screenings, transgenic larch calli were obtained^13,14^. Then, the transgenic embryogenic cell line was induced to develop into somatic embryos via somatic induction media, after which the somatic embryos were induced to develop into transgenic larch seedlings by 1/2MS rooting media^15^.

### Acquisition and detection of resistant calli

The DNA of wild-type and transgenic embryogenic calli was extracted, and the following full-length primers were used: *LoHDZ2-F* (ATGGAAGAGATGAAGAACAAGCA) and *LoHDZ2-R* (TTAGCAAGCTGCAGACTGTTGG). PCR detection showed that the amplified fragment was approximately 1000 bp. The PCR reaction mixture comprised 2 µL of template DNA, 1 µL of upstream and downstream primers, 1 µL of EasyTaq^®^ DNA polymerase, and 20 µL of P-mother liquor (P-mother liquor was prepared by mixing the following reagents: 10 × *EasyTaq*^®^ buffer:2.5 mM dNTPs:H_2_O = 5:3:2). The reaction procedure was as follows: 94 °C for 3 min; 35 cycles of 94 °C for 30 s, 60 °C for 30 s, and 72 °C for 1 min; 72 °C for 7 min; and then a pause at 16 °C.

RNA from transgenic larch calli was extracted and reverse transcribed into cDNA for qRT-PCR. Primer 5 software was used to design gene quantitative primers (*LoHDZ2*-RT-F, CTTGGCGTTGGTGTGTCTATG; *LoHDZ2*-RT-R, TGGGCATGAACCAAAGAAAC). An ABI7500 fluorescence qRT-PCR instrument was used, and a dissolution curve was generated. According to the standard procedure of the ABI7500 instrument. The reaction conditions were 94 °C for 30 s, 94 °C for 5 s, 60 °C for 15 s and 72 °C for 10 s. Forty cycles were required from the second step to the fifth step, and the difference among the three CT values was less than 1. Microsoft Excel 2016 was used for data analysis, with the -ΔΔCT formula used for calculations. Figures were constructed with GraphPad Prism5 software. The internal reference gene was *Larix* *olgensis* gene^16^ (the gene number on the NCBI database is MF278617.1) named *LoB80280.* The wild-type was used as a control. The expression of *LoHDZ2* in the transgenic calli was analyzed on the basis of the qRT-PCR results.

In terms of growing conditions for GUS-stained plants, the transgenic resistant embryogenic cell line was cultured in 1/10-BM media for 10 days. With respect to GUS staining, tissue from resistant plants was removed and immersed in an appropriate amount of X-Gluc (Sigma) staining solution, which was subjected to a vacuum until there were no bubbles in the staining solution; afterward, the tissue was incubated at 37 °C for 12–16 h^17^. GUS staining was observed under an Olympus microscope BX51 (Japan). Four transgenic lines were selected, and 0.6 g of each transgenic line was used for GUS staining, which was repeated 3 times. Wild-type tissues were used as negative controls to observe the color development of the calli.

### RNA-Seq analysis

#### Transcriptome sequencing and bioinformatics analysis

The total RNA of three transgenic cell lines (OE1, OE2 and OE4) and the wild-type callus sequencing samples was extracted and sent to Lianchuan Biotechnology Company for total RNA detection and HiSeq sequencing. The method was the same as that in Jianzhong Hu's study on the Arabidopsis transcriptome^18^.

Clean reads were obtained from the data after filtering. The original sequencing data clean reads were assembled de novo into a unigene sequence set by using Trinity assembly software. Mapping data were obtained by comparing the unigenes with the clean reads for library quality evaluation^19^. Differential gene expression analysis, functional annotation and enrichment analysis of the differentially expressed genes were carried out according to the gene expression differences between different sample groups^20^.

### Verification based qRT-PCR

Taking the cDNA of the transcriptome sample returned by the company as the template, 10 genes (5 upregulated genes and 5 downregulated genes) were selected from the differentially expressed genes revealed by the transcriptome sequencing for data verification. qRT-PCR primers were designed by Primer 5.0. The internal reference gene used was *LoB80280* (the gene number in the NCBI database is MF278617.1). The quantitative primers of the 10 differentially expressed genes are shown in Table 1. The method used was the same as that above.

**Table 1:** Quantitative primers used for qRT–PCR-based verification.

Gene_ID	Forward and reverse primers (5′–3′)	
TRINITY_DN29143_c1_g1	ACAGCGTCTCCGACCTGAT	ACTCCCCAATGTCAAGCACTTC
TRINITY_DN27466_c0_g1	ATTATCATTTCTGTTTGCCTGAC	TCGTGAACAGTTTGCAGTATGTG
TRINITY_DN24328_c0_g3	GGATCTAGCCAAATATCATCCCAC	TTCTTGACTGAAGTGCCTTGTGA
TRINITY_DN32335_c0_g2	AAGGAACAAGTAGCAACACTCAAAA	TCATTTGTTATTTCTTCAACCCTCT
TRINITY_DN25974_c0_g2	TTTTAGATCCTGTTCAACCAAATAAC	ATAAAGACCCAAATCCGAAGACA
TRINITY_DN36257_c2_g9	TTTGAATCTATGAGGCGAGTG	CAGCGTTTGCAGTATGAAGGT
TRINITY_DN24407_c0_g8	ATAGGCCCCAACAACTTAATG	GCTGTGCTCTTCACCTTGATAAT
TRINITY_DN24036_c0_g1	ATGGGTTCTCAAGTTATTGCA	AATTATTAGTCGGCACAGGGA
TRINITY_DN33840_c0_g2	CTGTTAGCGATTCTGATGGCC	TTGCCCACTTCCCTTACCC
TRINITY_DN28711_c0_g5	GAACTCAACGCCCACCAGC	CGTATGGTGAAACCATTTCGC
*LoB80280*	GCCGTGCTGCTGGATAATGAGG	TGTCTGGAACTCAGTCACATCAACG

### Growth of and gene expression in transgenic larch calli under different treatments

Fresh wild-type and transgenic embryogenic callus lines growing for 10 days were selected and cultured on 20% PEG_6000_ and on proliferation media supplemented with 200 mM NaCl (1/10-BM) (prepared according to the ratio of 1:20 g/mL). The materials were removed at 0 h, 24 h, 48 h, 72 h and 96 h after stress treatment, frozen in liquid nitrogen, and stored at − 80 °C for subsequent tests. Each treatment included three replications, with three callus lines placed in each plate, and the fresh weight of each callus was 0.6 g (three replications per plate). Then, the RNA of the treated transgenic calli was extracted and reverse transcribed into cDNA for qRT-PCR. The specific method used was the same as that above.

The samples were stored at − 80 °C, and the contents of peroxidase (POD), malondialdehyde (MDA) and superoxide dismutase (SOD) were determined. A Suzhou Grace Biotechnology Co.Ltd. test kit (spectrophotometer method) was used according to the manufacturer’s instructions.

### Statement

I declare that all test methods are carried out in accordance with the relevant guidelines, and there is no violation of the relevant provisions.

## Results

### Genetic transformation of larch

**Figure 1:**
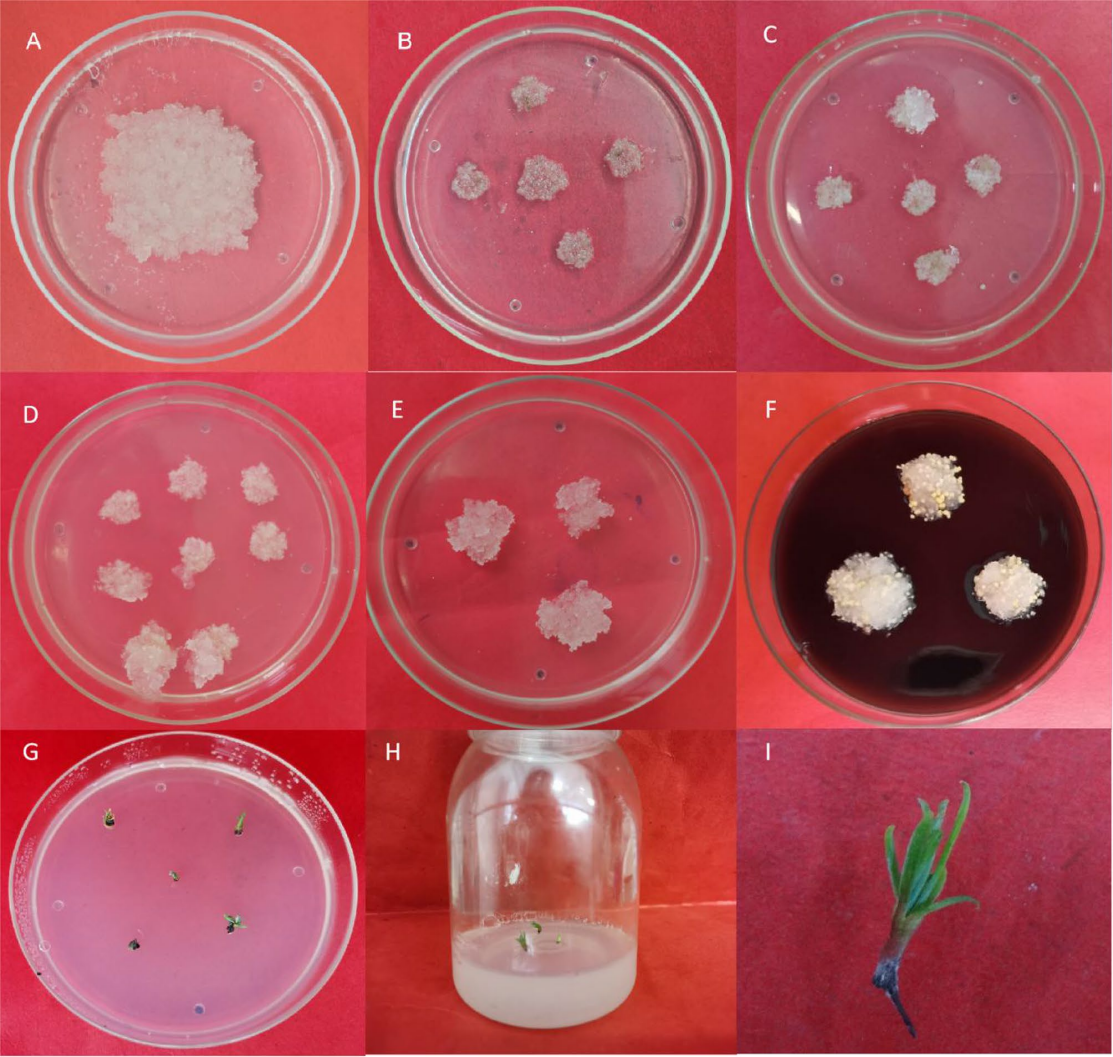
Process of transgenic callus induction (**A**) Genetic transformation of larch calli; (**B**) First screening; (**C**) Second screening; (**D**) Third screening; (**E**) Transgenic callus transition culture; (**F**) Somatic embryo induction of transgenic larch. (**G**–**I**) Somatic embryo induction rooting process.

**Figure 2:**
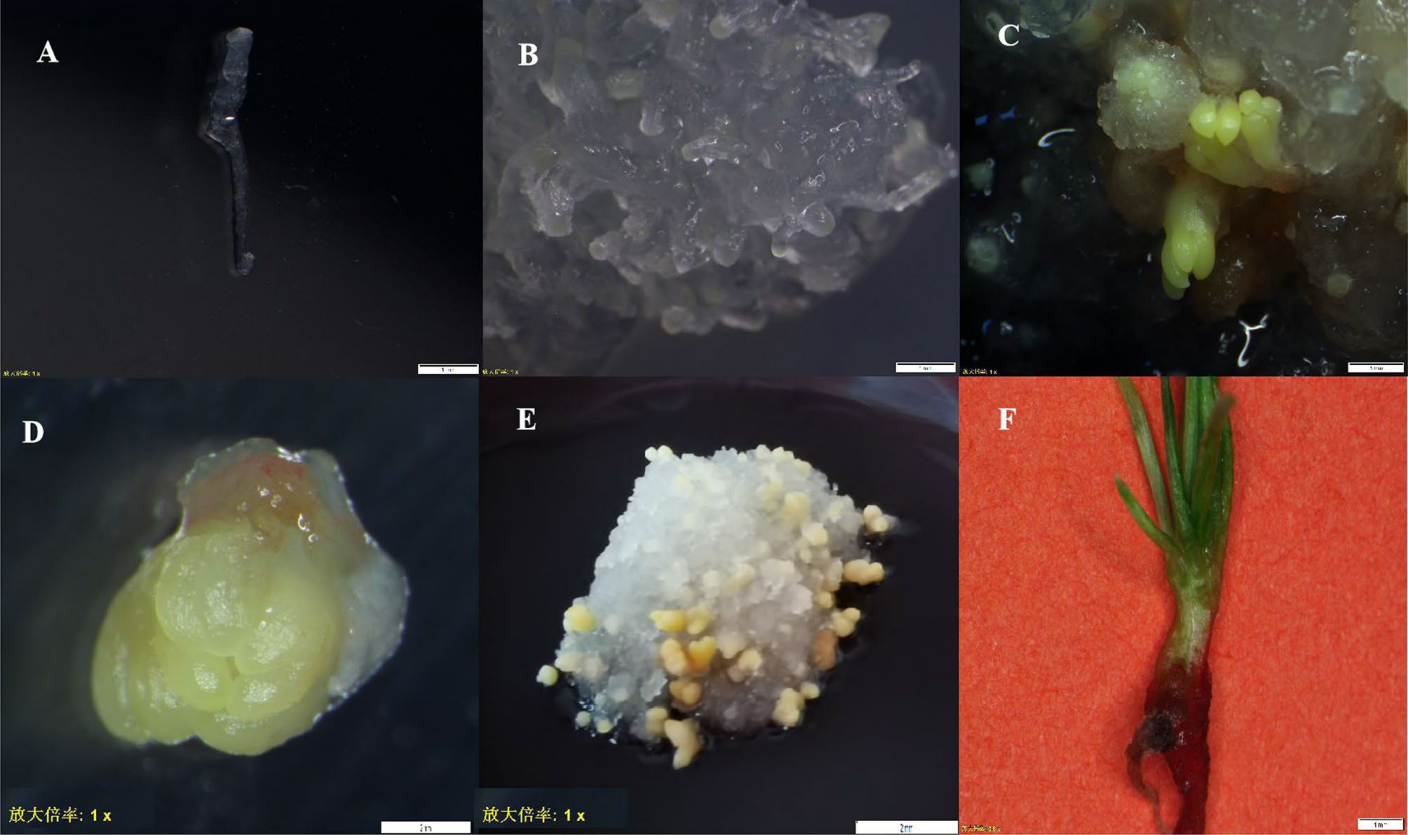
Microscopic view of larch somatic embryo maturation and germination. (**A**) Mature somatic embryos of transgenic larch; (**B**) Somatic embryo (×8 microscopy); (**C**) Larch seedlings that have differentiated for 20 days; (**D**) Transgenic larch seedlings after 30 days of growth in differentiation treatment.

Embryogenic calli on 1/10-BM proliferation media were selected for genetic transformation of larch. The genetically transformed calli were cocultured for approximately 2 days and then transferred to culture media consisting of 500 mg/L Cef for strict sterilization. After three separate cultures on the screening media, five resistant cell lines were obtained. The resistant cell lines were then cultured in proliferation media for 10 days, after which they then in 1/4-BM transition media for 10 days. Afterward, the calli were placed in somatic embryo induction media for somatic embryo induction to obtain larch transgenic somatic embryos (Fig. 1). Then, the somatic embryos were placed in 1/2MS rooting media to obtain transgenic larch seedlings, as shown in Fig. 2.

### Molecular detection of transgenic *LoHDZ2* calli

**Figure 3:**
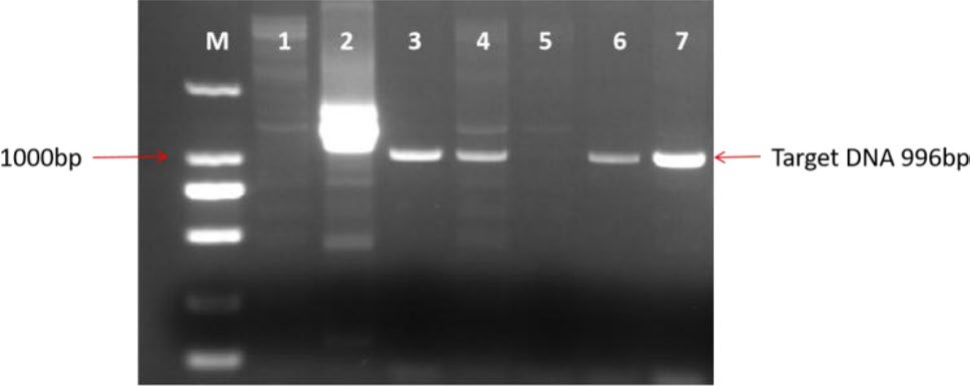
PCR-based detection of VB191103-*LoHDZ2* *t*ransgenic callus. M: 2000 bp DNA molecular marker; 1: negative control; 2: positive control; 3–7: transgenic strain. The image is cropped,full-length blots/gels are presented in Supplementary Fig. 3.

Five transgenic callus cell lines growing on 1/10-BM were randomly selected to extract plant genomic DNA. The DNA was subsequently used as template for PCR detection. VB191103-*LoHDZ2*::GUS plasmids were used as positive controls, and the wild-type was used as a negative control. The PCR detection results of VB191103-*LoHDZ2*::GUS transformed embryogenic calli are shown in Fig. 3. The results showed that four transgenic calli produced bands at corresponding positions, which preliminarily showed that VB191103-*LoHDZ2*::GUS had been integrated successfully into the larch genome.

**Figure 4:**
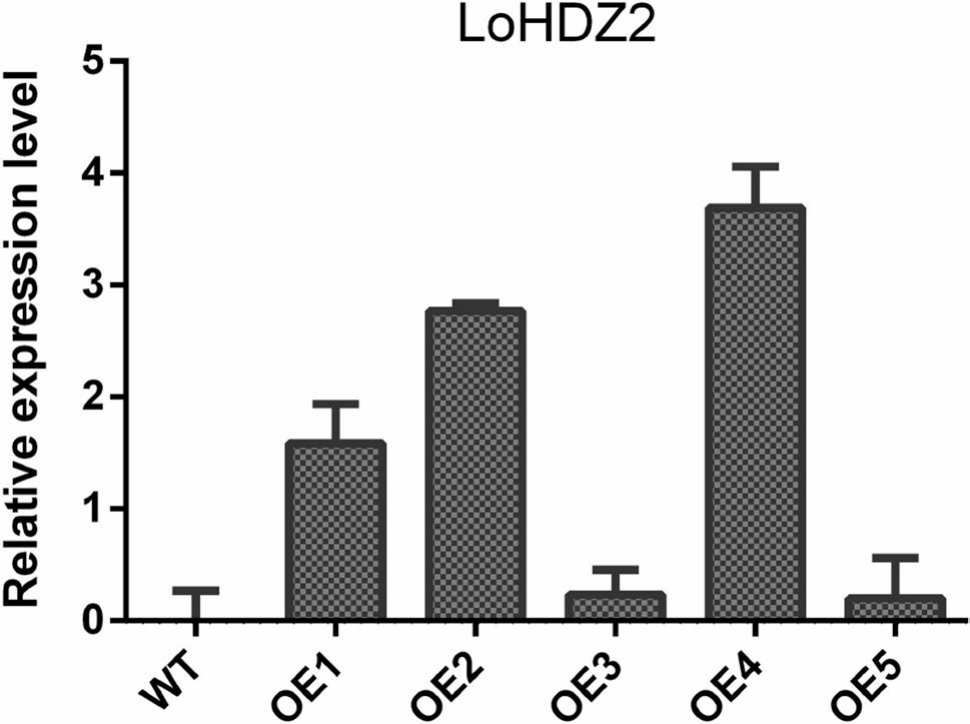
QRT-PCR-based detection of different embryogenic lines of transgenic calli. *WT* wild-type callus, *OE1–OE4* different transgenic lines.

The five above mentioned resistant callus cell lines growing on 1/10-BM media and one wild-type callus cell line were collected, and their RNA was extracted and then reverse transcribed into cDNA. The cDNA was used as a template for qRT-PCR-based detection, and wild-type cDNA was used as a control. The results showed that the expression levels of transgenic lines OE1, OE2 and OE4 were higher, approximately 1.9, 2.9 and 3.6 times higher, respectively, than that of the wild-type cell lines(Fig. 4).

**Figure 5:**
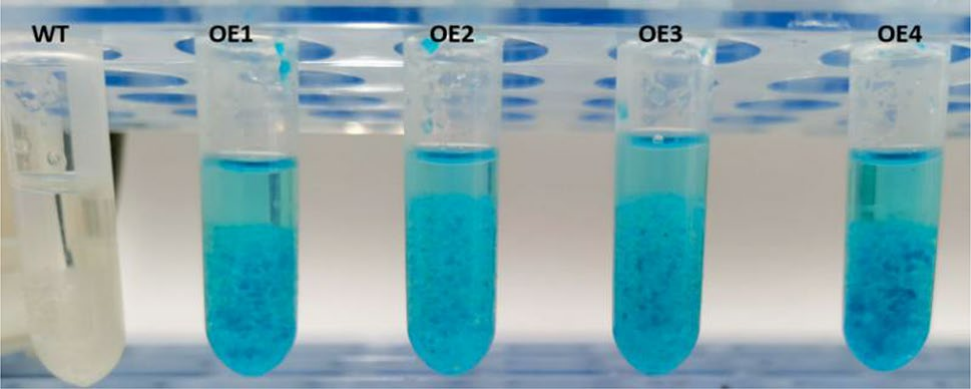
GUS staining of transgenic and wild-type calli. *WT* wild-type callus, *OE1–OE4* calli of different transgenic lines.

**Figure 6:**
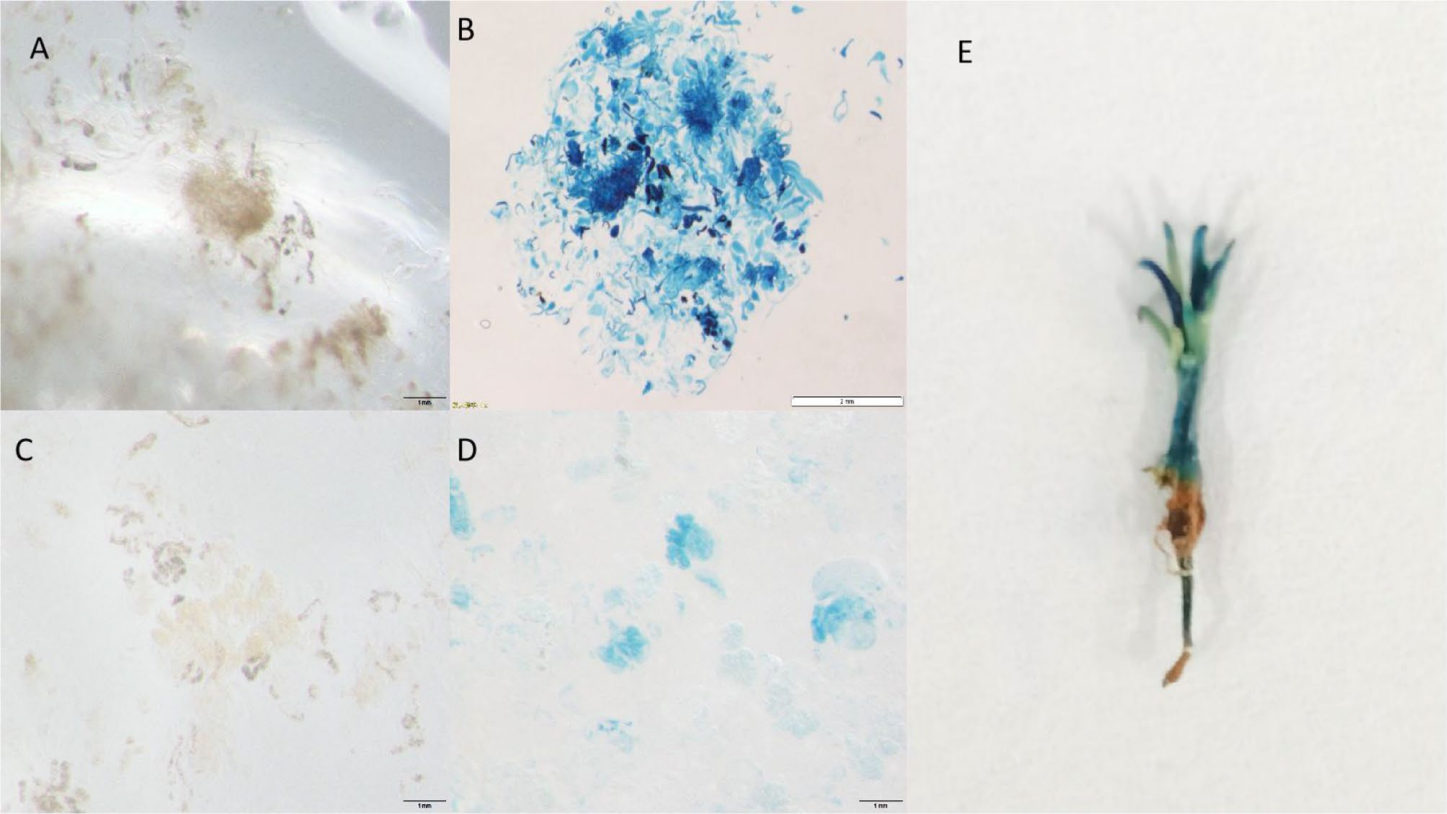
Microscopy results of GUS staining of transgenic and wild-type calli. (**A**) GUS staining of wild-type calli (×1 magnification); (**B**) GUS staining of transgenic calli (×1); (**C**) wild-type calli subjected to GUS staining (×10); (**D**) GUS staining of transgenic calli (×10); (**E**) GUS staining of transgenic plantlet.

To further confirm that VB191103-*LoHDZ2*::GUS was integrated into the resistant calli, GUS staining solution was applied to wild-type larch calli and resistant calli. The results showed that four of the transgenic calli turned blue, while the wild-type callus in the control group exhibited no color change, indicating that the recombinant VB191103-*LoHDZ2*::GUS plasmid had been successfully integrated into the larch calli (Figs. 5, 6).

According to the detection of the DNA and RNA in the transgenic cell lines and the analysis of the GUS staining results, the OE1, OE2 and OE4 transgenic cell lines were ultimately selected as follow-up research materials.

### RNA-seq analysis

#### Unigene functional annotation

The assembled unigenes do not have corresponding functional annotations, so we added corresponding functional annotations using Diamond, a new comparison software similar to BLASTX. Because the sequences of similar functional genes (nucleic acid sequences or protein sequences) are highly conserved among different species, we selected six authoritative databases, namely, the NCBI NR, GO, KEGG, Pfam, SwissProt and eggNOG databases^21^ (Table 2). The off-line data obtained by sequencing needs to be preprocessed. Lianchuan biological uses cutadapt to remove the sequencing connector, and using fqtrim to filter out the unqualified sequences to obtain clean data (Table 3). Adopt the strategy of mixed assembly of all samples, and finally normalize all samples to obtain UniGene (Unigene is the only gene obtained after de redundancy and de duplication of all genes in each sample). Next, the assembly quality of these unigenes will be evaluated, including the length, GC content and N50 of UniGene. N50 can be used as a criterion to judge the splicing results of genome and transcriptome, which refers to the length when it reaches half of the total length after arranging all the assembled results from high to low (Table 4).

**Table 2:** Unigene annotation statistics.

DB	Number	Ratio (%)
All	68,288	100.00
GO	23,199	33.97
KEGG	18,469	27.05
Pfam	21,376	31.30
SwissProt	20,152	29.51
eggNOG	25,781	37.75
NR	27,746	40.63

**Table 3:** The summary statistics of the RNA-seq data.

Sample	Raw_Reads	Raw_Bases	Q20%	Q30%	GC%
*LoHDZ2A*	41,790,946	6.27G	98.62	95.43	45.01
*LoHDZ2B*	40,767,766	6.12G	98.59	95.38	45.02
*LoHDZ2C*	40,187,840	6.03G	98.62	95.40	45.08
WT1	36,936,438	5.54G	98.64	95.51	45.21
WT2	40,609,562	6.09G	98.59	95.36	45.11
WT3	45,483,110	6.82G	98.55	95.27	44.97

**Table 4:** Trinity assembly results.

Index	All	GC%	Min Length	Median Length	Max Length	Total Assembled Bases
Transcript	154,096	41.55	201	585.00	16,122	156,170,579

### Verification based qRT-PCR

**Figure 7:**
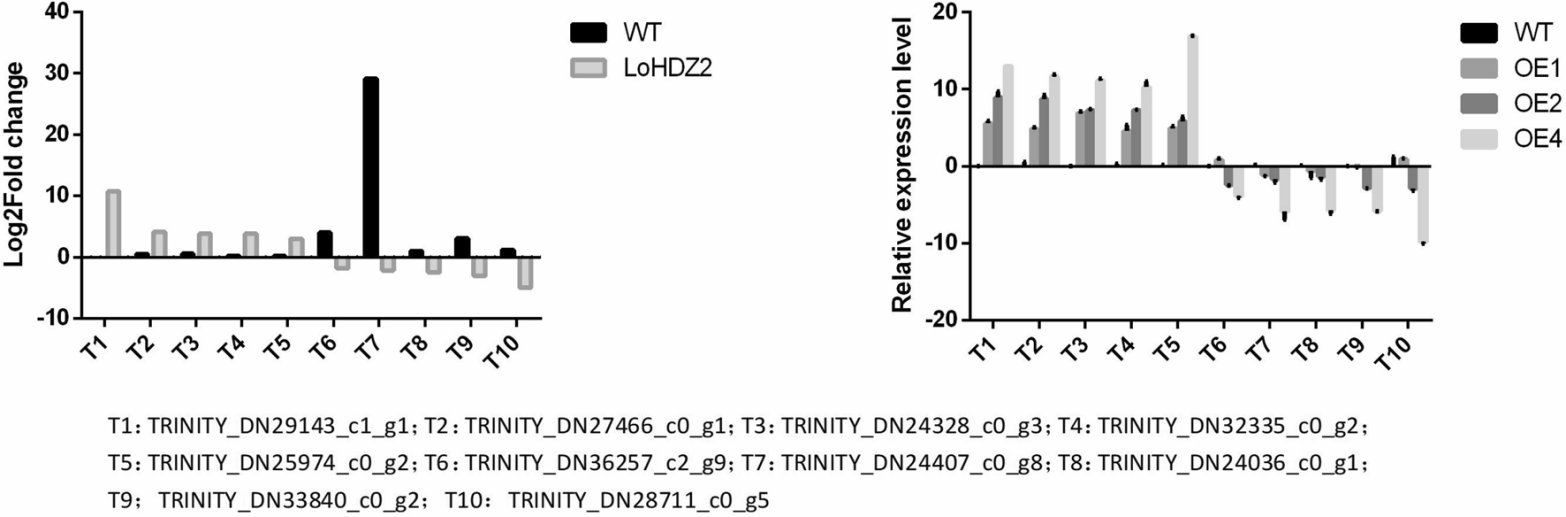
Gene expression according to qRT-PCR. The image is cropped,full-length blots/gels are presented in Supplementary Fig. 5.

To verify the accuracy and reliability of the transcriptome data, 10 differentially expressed genes (5 upregulated and 5 downregulated ones) were selected for qRT-PCR based verification. The results show that these genes have different expression fold changes, as detected by RNA-Seq and qRT-PCR (Fig. 7). The correlation coefficient between RNA-Seq and qRT-PCR was 0.942, p < 0.01. This may be because the sensitivity of the two detection methods is different. Nonetheless, the expression trends are consistent, indicating that the transcriptome data are reliable and can be used for further gene functional analysis.

### Screening of differentially expressed genes

**Figure 8:**
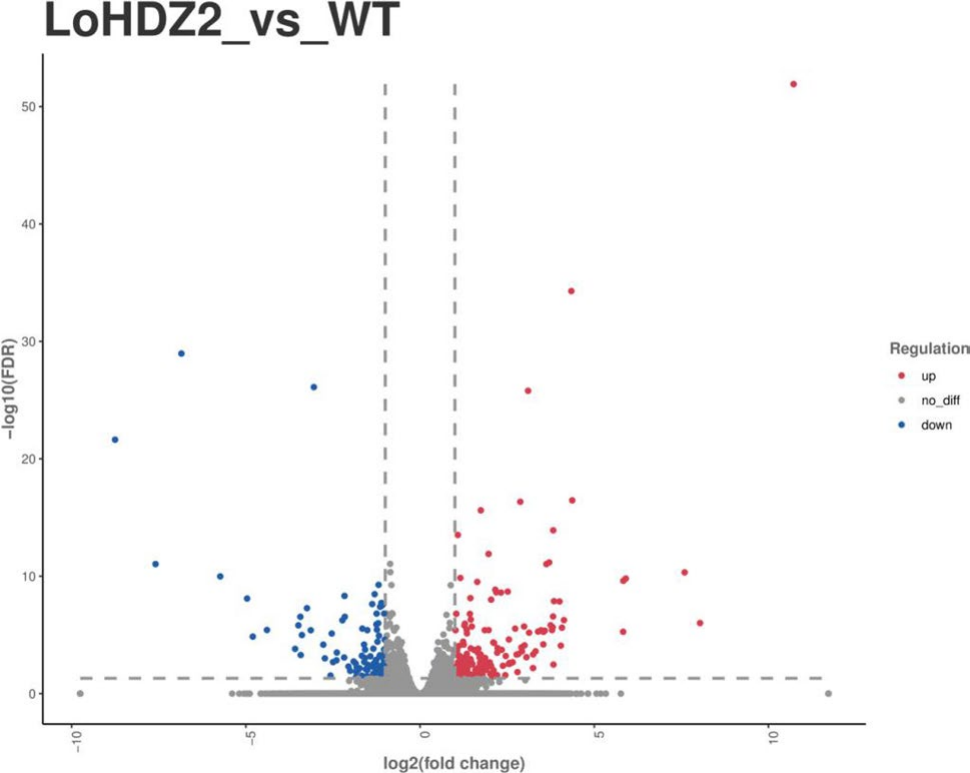
Different expression gene venn diagram in different samples.

In this study, DESeq2 was used to screen differentially expressed genes. The differentially expressed genes among different varieties were screened on the basis of their expression fold change (|log2(fold change)|> 1) and significance level (p < 0.05). The results showed that there were 304 differentially expressed genes in transgenic larch cell lines compared with wild-type cell lines: 167 upregulated differentially expressed genes and 140 downregulated differentially expressed genes (Fig. 8).

### Cluster analysis of differentially expressed genes

**Figure 9:**
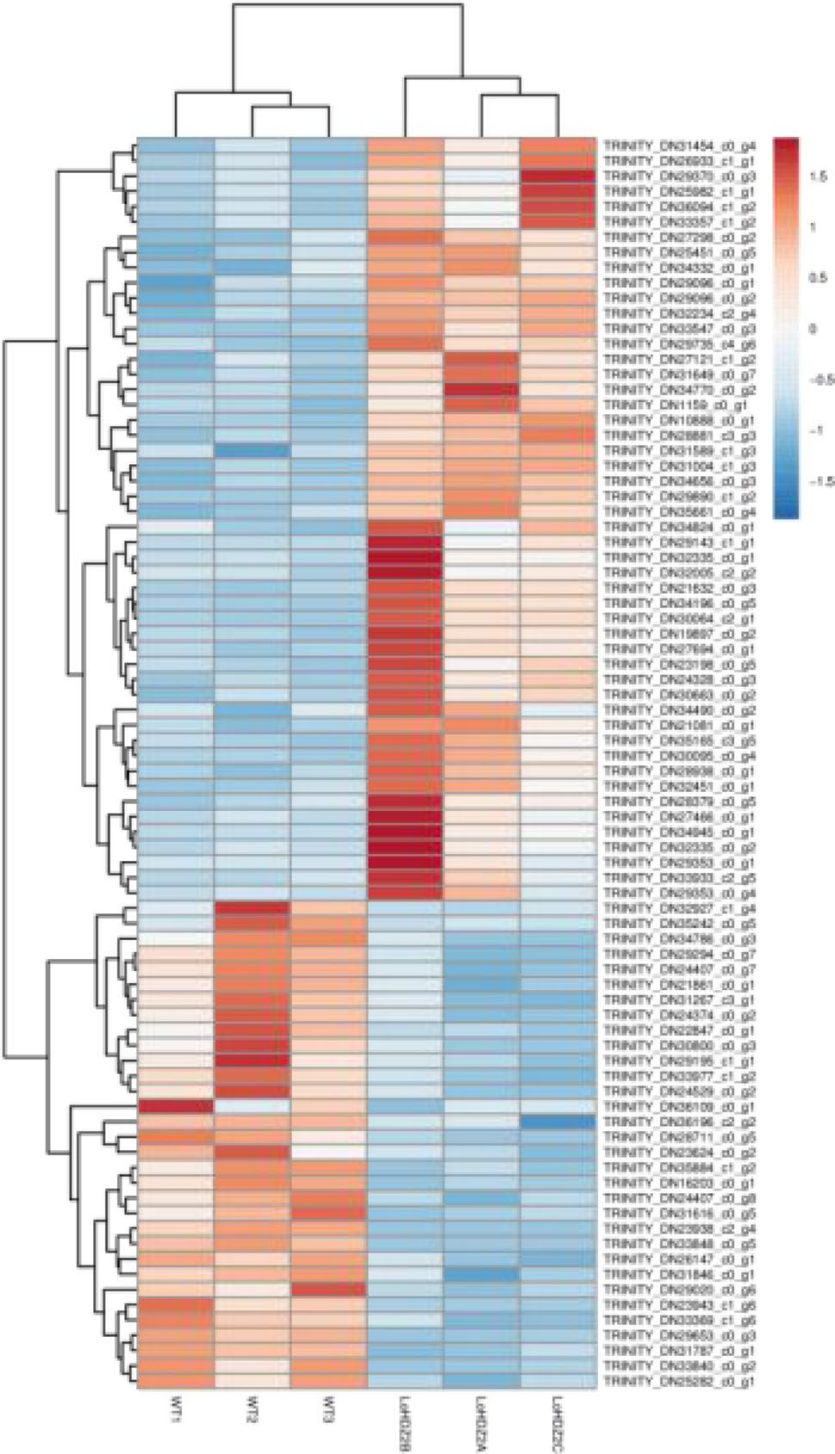
Different expression gene heatmap-top50.

To compare the clustering patterns of the differentially expressed gene expression profiles of the transgenic and wild-type calli, a clustering heatmap of differentially expressed genes was constructed for the genes with a large variance in expression in different samples (Fig. 9). The results showed that compared with the wild-type resistant calli, the expression of many genes in the transgenic resistant calli has changed; the number of upregulated genes especially changed, which was approximately 176. There were 140 downregulated genes, indicating that the overexpression of *LoHDZ2* has a positive regulatory effect on downstream genes. The heatmap shows that the transcriptome sequencing of the three biological replicates of each sample have good consistency, indicating that the sequencing data are relatively reliable.

To further explore the function of *LoHDZ2*, the differentially expressed genes with the largest variance in the transgenic and wild-type samples were used to construct a cluster heatmap of differentially expressed genes, and 46 upregulated genes and 30 downregulated genes with large expression fold changes were selected for NCBI BLAST (https://blast.ncbi.nlm.nih.gov/Blast.cgi). When the results were compared with the information in the various aforementioned databases, relevant annotation and species information was obtained.

Among the 46 differentially expressed genes, including 22.0 kDa class IV, heat-shock protein-like 22.0 kDa IV, heat stress transcription factor A-1-like, heat-shock proteins, and heat-shock cognate 70 kDa protein, most have been annotated in broad-leaved tree species^22^. The annotated LRR receptors, such as the serine/threonine protein kinase FLS2 LRR receptor and serine/threonine protein kinase FLS2, may inhibit cell proliferation and stimulate extracellular matrix synthesis depending on the cell type. In addition, the xyloglucan galactosyltransferase GT11, which affects the internal mechanisms of plant growth and development, was also annotated^23^.

Among the 30 downregulated differentially expressed genes, poly[ADP ribose] polymerase 3 isoform X2, a late-embryogenesis abundant protein, was identified^24^. Moreover, this protein plays an important role in plant growth and development, in disease resistance, and in response to hormones and stress^25^ and is involved with E3 ubiquitin-protein ligase SINAT2-like protein.

### GO functional annotation and enrichment analysis

GO functional annotation was carried out for the differentially expressed genes in the different samples. The differentially expressed genes were annotated to the three different GO classification categories: cellular components, biological processes and molecular functions. The number of differentially expressed genes between the different samples and the wild-type is annotated to the three branches. After GO functional classification annotation, 769 genes were annotated. Among them, 243 gene pathways were enriched in cellular components, 194 molecular function pathways, and 334 genes were annotated to biological process pathways. The enriched differentially expressed genes and their information are shown in Table 5.

**Table 5:** Statistics of differentially expressed genes enriched in GO classification terms.

GO category	GO enrichment information	Number of differentially expressed genes	Percentage (100%)
Cellular component	Nucleus	39	16.0
	Cytoplasm	31	12.8
	Chloroplast	17	7.0
	Cytosol	15	6.2
	Plasma Membrane	13	5.3
	Integral Component of Membrane	13	5.3
	Mitochondrion	8	3.3
	Cell wall	6	2.5
	Plasmodesma	6	2.5
Molecular function	Protein binding	20	10.3
	DNA binding	8	4.1
	Metal ion binding	8	4.1
	ATP binding	7	3.6
	Molecular function	7	3.6
	Hydrolase activity	5	2.6
Biological process	Regulation of transcription, DNA-templated	23	6.9
	Response to heat	11	3.3
	Biological process	7	2.1
	Response to hydrogen peroxide	6	1.8
	Response to cadmium ion	6	1.8
	Response to high light intensity	5	1.5
	Metal ion transport	4	1.2
	Lipid catabolic process	4	1.2
	Response to stress	4	1.2
	Response to wounding	4	1.2
	Multicellular organism development	4	1.2
	Defense response to bacteria	4	1.2
	Response to abscisic acid	4	1.2
	Defense response	4	1.2

In the cellular component pathway, the most abundant genes were associated with terms such as nucleus (39), cytoplasm (31), chloroplast (17), cytosol (15), plasma membrane and integral components of membrane (16), mitochondria (8), and cell wall and plasmodesmata (6). In the molecular function pathway, most (43) genes were related to binding proteins, followed by catalytic activity and molecular function, so binding and catalytic activity are the main molecular pathway processes. Among the pathways involved in biological processes, the largest number of enriched genes was associated with the response to various self-processes (40), followed by transcriptional regulatory processes (23) and then metabolic processes (12). These differentially expressed genes screened by GO enrichment analysis are involved in biological processes such as larch metabolism and response to stimuli. Moreover, these genes may be closely involved in regulating the response of larch to external stimuli and larch growth processes.

### KEGG functional annotation and enrichment analysis

**Figure 10:**
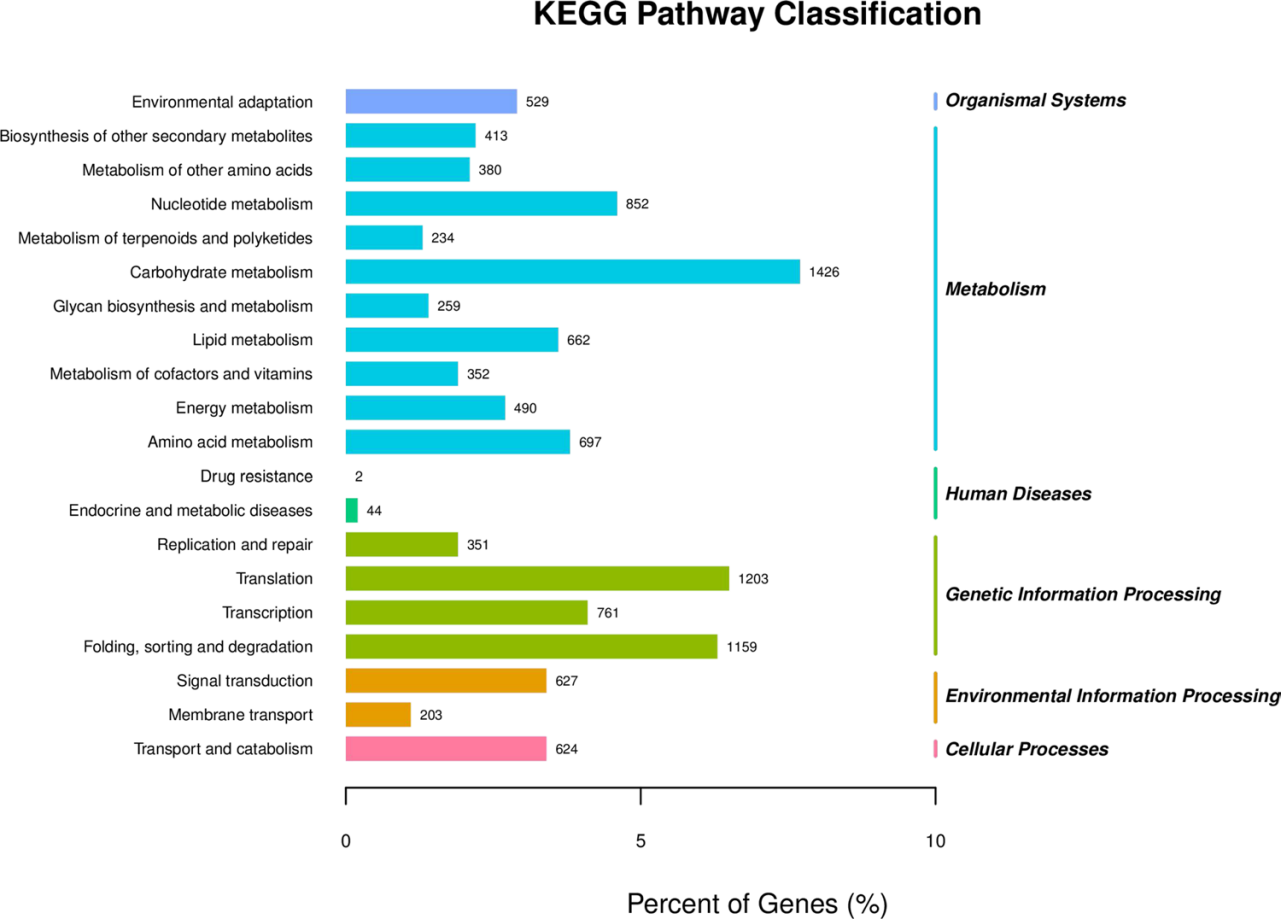
KEGG was enriched in transgenic and wild-type cell line samples.

The annotation results of the differentially expressed genes were classified according to type of KEGG pathway. The results showed that the differentially expressed genes were enriched in 20 metabolic pathways (Fig. 10). The metabolic pathways were divided into five pathway types, namely, organic systems, environmental information processing, metabolism and genetic information processing and cellular processes. Among them, the pathways enriched in metabolism were the most enriched, including 10 metabolic types, accounting for 50% of the whole enrichment pathway; these pathways mainly included those associated with carbohydrate synthesis, nucleotides, amino acid metabolism, lipid metabolism and energy metabolism. This shows that, on the basis of the premise of normal biological growth, different genes may participate in adaptations associated with plant growth and development via various metabolic pathways.

The genetic information processing pathway had the largest number of genes, including genes related to genetic information processes involving DNA transcription, protein translation, protein folding, classification and degradation. There are two types of environmental information processing pathways: signal transduction and membrane transport. The other two pathways have only one type, namely, environmental adaptation, transportation and catabolism. A variety of metabolic pathways involved in the growth, development and stress resistance of larch, and these metabolic pathways are coordinated by a variety of transcription factor-encoding genes. However, the specific cooperation needs to be further studied and verified.

### Determination of physiological and biochemical indices under different treatments

#### Effects of different stress treatments on the POD activity of transgenic calli

POD activity reflects the ability of plants to scavenge H_2_O_2_ and other reactive oxygen species. POD is an enzyme closely related to energy and respiratory metabolism, and POD activity has effects of antioxidation and delaying aging^26^. The higher the activity of POD is stronger than the physiological metabolism and antioxidant capacity of plant tissue, which can accelerate the removal of active oxygen such as H_2_O_2_ and lead to better adaptability to adverse conditions^27^.

**Figure 11:**
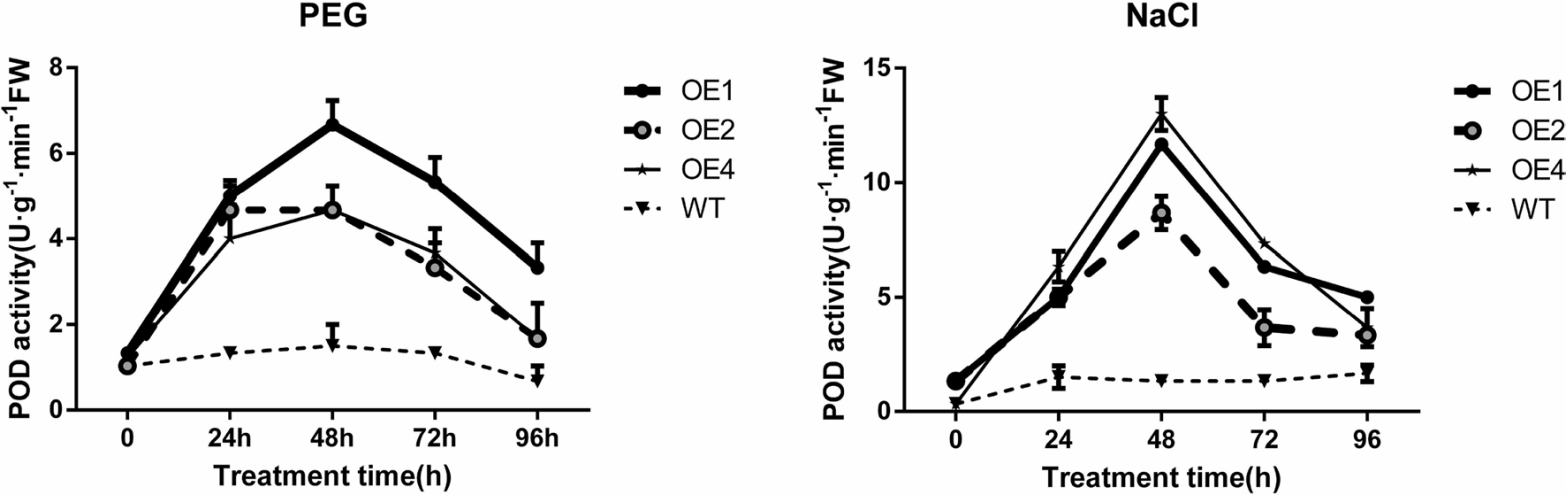
POD activity of wild-type and different transgenic cell lines under different treatment.

It can be seen in Fig. 11, Under PEG_6000_ simulated drought stress, the POD activity of wild-type calli was very weak with increased stress duration and was always lower than that of the transgenic cell line. The transgenic cell lines showed a trend of "first decreasing, then increasing and then decreasing again", and the change trend of the three transgenic cell lines was very consistent. When the calli were treated for 96 h, the values of OE2 and OE4 were the same as those of the wild-type calli, but the value of OE1 was higher than that of the wild-type and the other two transgenic cell lines. These results show that under drought stress, the POD enzyme activity of the transgenic cell line is stronger than that of the wild type, which means that POD can effectively remove the peroxide produced by stress, increase drought resistance.

Under NaCl stress, the POD activity of the wild-type calli was stable with increasing stress duration, but it was lower than that of transgenic cell line. The transgenic cell lines generally showed the trend of "increasing first and then decreasing", and the value was the highest at 48 h of stress treatment. These results indicated that 48 h of treatment is a node, and the expression trend of the three transgenic cell lines was very consistent, indicating that the transgenic cell lines have a certain degree of salt tolerance.

#### Effects of different stress treatments on the MDA content of transgenic calli

**Figure 12:**
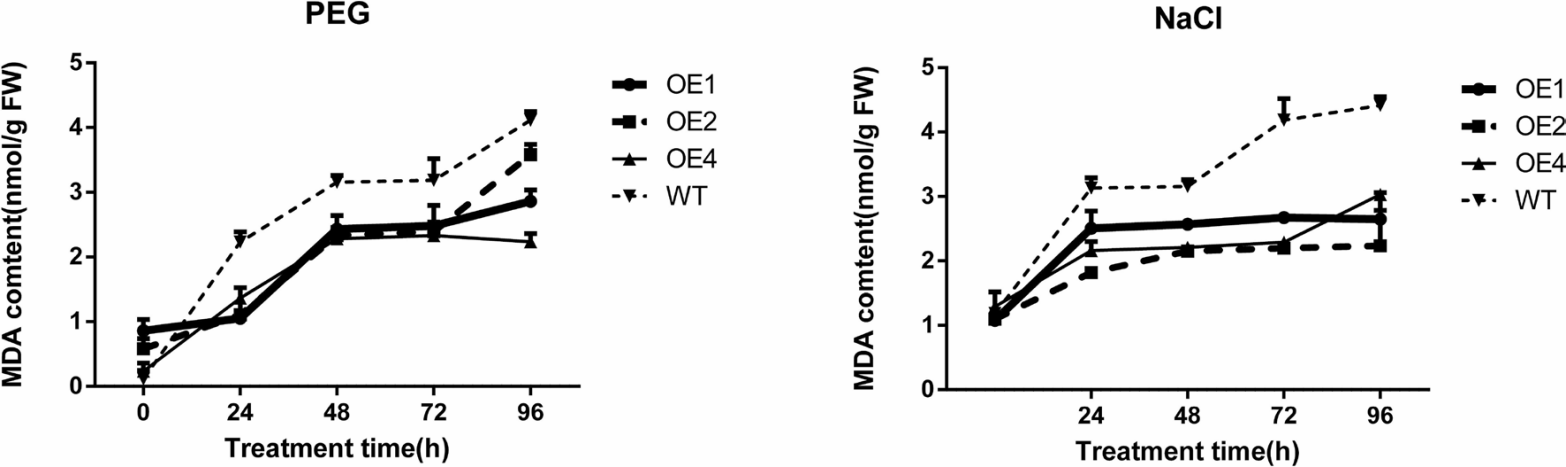
MDA content of wild-type and different transgenic cell lines under different treatments.

It can be seen in Fig. 12, MDA is the main product of membrane lipid peroxidation in plants under stress^28^, and its amount can indirectly reflect the degree of oxidative damage to plants caused by stress. The higher the MDA content is, the greater the degree of oxidative damage^29^. At the same time, the protective enzyme system composed of SOD (superoxide dismutase) and POD can not only remove excess reactive oxygen species over time, but also remove excessive amounts of MDA, which can reduce membrane lipid peroxidation and protect membrane structure^29^.

Under 20% PEG_6000_ simulated drought stress, the MDA content of the wild-type calli increased first and then decreased with the prolonging of stress and reached the maximum at 48 h. The transgenic cell lines also showed a similar trend, but the MDA content reached the highest after 48 h of stress. Moreover, the MDA content of the transgenic cell lines was lower than that of the wild-type cell lines after 24 h and 48 h of stress, and the accumulation degree of harmful substances was low, indicating that the degree of oxidative damage of the transgenic cell lines was less than that of the wild-type cell lines.

Under NaCl stress, the MDA content of wild-type and transgenic cell lines decreased with the extension of stress time. In general, the MDA content of the transgenic cell lines was lower than that of the wild-type cell lines, indicating that the degree of oxidative damage of the transgenic cell lines was lower than that of the wild-type cell lines.

#### Effects of different stress treatments on the content of superoxide dismutase (SOD) in calli

**Figure 13:**
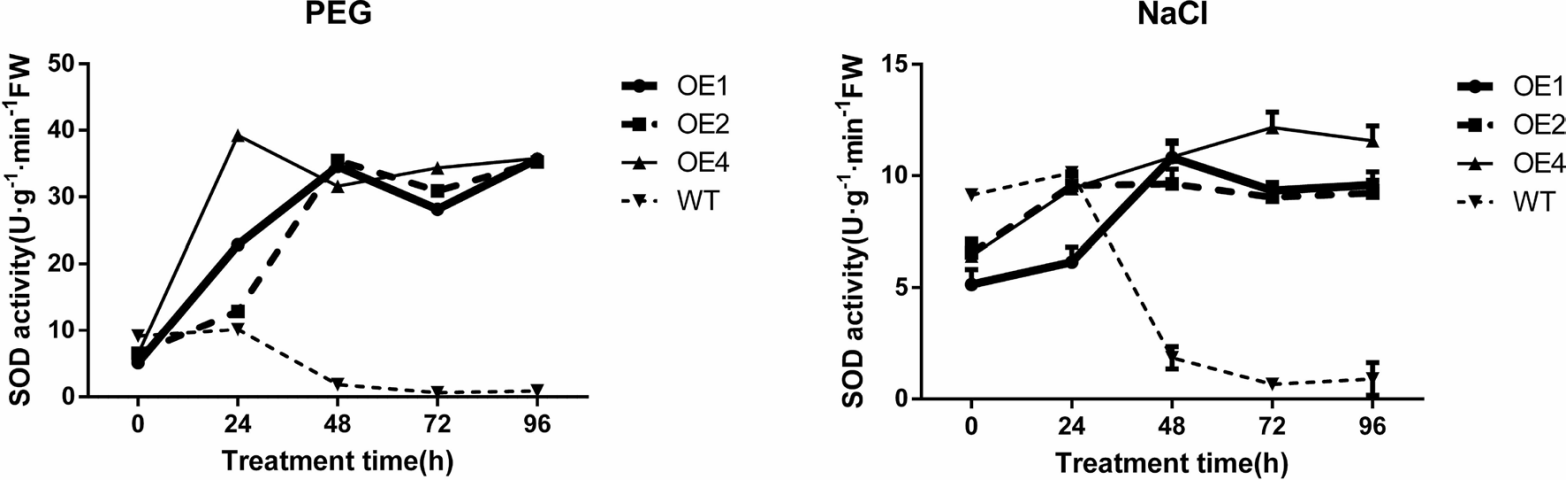
SOD content of wild-type and different transgenic cell lines under different treatment.

It can be seen in Fig. 13, as an important antioxidant enzyme, SOD catalyzes the disproportionation of superoxide anion radicals to produce hydrogen peroxide and oxygen to remove reactive oxygen species and ultimately improve the ability of plants to resist drought stress^13^.

Under PEG_6000_ simulated drought stress, the SOD content of the wild-type calli showed a trend of "increasing first and then decreasing" with the extension of stress duration, peaking at 24 h. The transgenic cell lines also expressed a similar trend, in which the SOD content of OE1 and OE2 reached the maximum after 48 h of treatment, while that of OE4 reached the maximum after 24 h of treatment. Nonetheless, both of these maximum contents were higher than the those of the wild-type cell lines, indicating that the drought resistance of the transgenic cell lines was enhanced.

Under NaCl stress, the SOD content of wild-type cell lines decreased linearly after 24 h of stress treatment and decreased continuously until 96 h of treatment. The SOD content of the three transgenic cell lines was higher than that of the wild-type, and the SOD content of the three transgenic cell lines was relatively high, indicating that the transgenic cell lines had greater salt stress resistance than the wild-type cell lines did.

## Discussion

At present, there are few reports on the successful genetic transformation of conifers such as larch, which is mainly due to the difficulty of foreign gene transformation and integration, imperfect regeneration systems and so on^30^. In this study, the embryogenic cell line of *Larix* *olgensis* was stably transformed by Agrobacterium-mediated genetic transformation, and a cell line overexpressing the *LoHDZ2* gene of the HD-Zip II subfamily was obtained. Furthermore, the transgenic cells were successfully induced to develop into somatic embryos and then into seedlings.

HD-Zip II subfamily genes are mainly involved in the light avoidance response and respond to changes in light quality^31^. Expression analyses have confirmed that some of these HD-Zip II genes are indeed regulated by auxin in wheat. Together, our results suggest that HD-Zip II subfamily transcription factors regulate plant development, possibly through the auxin pathway in plants^32^. In early research, the *LoHDZ2* was successfully transformed into tobacco, and transgenic tobacco was obtained. After determining the phenotypic characteristics and physiological and biochemical indices of the transgenic tobacco, researchers found that transgenic tobacco exhibited plant dwarfing, leaf enlargement and early flowering phenomena. It was thus preliminarily speculated that this gene may be related to plant growth and development. In the present study, transgenic and wild-type tobacco were stressed with PEG_6000_ and NaCl, and their physiological and biochemical indices were measured. According to the physiological and biochemical indices, the transgenic cell lines had stronger resistance to external stress than the wild-type cell lines did. Many studies have shown that there is a close relationship between plant growth and resistance. For example, Wu Yuepeng and others used transgenic technology to import stress resistance-related genes to obtain transgenic plants; these plants exhibited improved plant stress resistance to a certain extent, but there were also problems such as stunted growth and development and dwarfing^33^.

Li et al. studied the growth and development of poplar and *Arabidopsis* leaves, stems and adventitious roots and plant stress resistance by transforming poplar with *Ptowusa*, *Ptowox4a*, *Ptowox5a* and *Ptrhsp17.8*. They found that the growth of plants was affected, which were dwarf-like but exhibited increased plant resistance^34^. Perennial ryegrass with *DREB1A* gene (DREB) and perennial ryegrass with BADH-CMO gene (BC) showed different degrees of dwarfing. It was found that the leaf cells of transgenic plants and nontransformed plants were significantly shorter than those of the nontransformed plants, and the perennial ryegrass with the *DREB1A* gene was resistant to high temperature; thus, the *DREB1A* gene and the BADH-CMO gene improved the drought resistance of perennial ryegrass^35^. In response to biological stress, a gene silencing pathway, hormone-mediated signal transduction pathway and metabolic regulatory pathway usually function in plants. These response mechanisms are coordinated by hormone signals and other small molecular signals^31^.

HD-Zip genes can improve the activity of antioxidant enzymes and the accumulation of some soluble organic substances^36,37^. For example, transgenic pepper, cotton and poplar plants expressing *HDG11* have high levels of proline, soluble sugars, and antioxidant enzymes (SOD and CAT) under high concentrations of NaCl, which reduces the oxidative damage of plants and is conducive to tolerance to salt stress by regulating osmotic homeostasis. Moreover, *HDG11* transgenic plants show a low content of MDA. As an end-product of lipid peroxidation, the lower the MDA content is, the more helpful it is to maintain the homeostasis of membranes and proteins in vivo^6,38^. Plant growth regulatory networks are very complex and have mutual influences. Therefore, it is preliminarily speculated that *LoHDZ2* of the HD-Zip II subfamily may be involved in plant growth, metabolism and stress resistance.

Temperature stress includes high-temperature, low-temperature and severe variable-temperature stress^39^. HD-Zips are mostly studied under high-temperature and low-temperature stress^40^. Under these two stresses, most enzyme activities are decreased, resulting in abnormal biochemical reactions and possibly cell death^41^. The expression of some HD-Zip transcription factors can be induced under both high- an low-temperature stresses; for example, some genes of cucumber and potato are upregulated under these conditions^42^. In warm (20–30 °C), dry environments, *HaHB4* induced the expression of redox-related and heat-shock protein-coding genes in transgenic soybean, indicating the potential function of *HaHB4* in the heat resistance mechanism^43^. Studies have shown that heavy metals are inducers of lipid peroxidation. When plants are polluted with heavy metals, especially toxic heavy metals, these metals can disrupt the structure and function of membranes and affect plant metabolism. The content of some heavy metal elements such as iron (Fe) needs to be maintained at steady state for plants to develop normally. Athb1 negatively regulates the expression of cafer1 in response to iron by binding to MYB transcription factors, inhibiting the overexpression of iron-related genes and participating in the regulation of iron homeostasis^44,45^.

Because it is difficult to cultivate larch in vitro regenerators, difficult to root, and long growth cycle, a large number of seedlings cannot be obtained in a short time^46^. Therefore, in order to clarify the function of *LoHDZ2*, *LoHDZ2* was transformed into model plant tobacco^47^, and transgenic tobacco was successfully obtained. After cultivating transgenic tobacco plants, the phenotypic characters and physiological and biochemical indexes of different transgenic lines were determined. The results showed that transgenic tobacco showed the phenomenon of plant dwarfing, leaf enlargement and early flowering. Transgenic and wild-type tobacco were stressed with PEG_6000_ and NaCl, and their physiological and biochemical indexes were measured. According to the analysis of the measurement results, the transgenic cell line has stronger ability to resist external stress than the wild-type cell line^48^. Plants usually have gene silencing pathway, hormone mediated signal transduction pathway and metabolic regulation pathway in response to biological stress. These response mechanisms are coordinated by hormone signals and other small molecular signals^49^.

The HD-Zip II subfamily member *HAT1* inhibits resistance against yellow mosaic virus (CMV) by inhibiting the expression of defense-related genes in Arabidopsis, such as PR1, PR2, and the ROS-related drug-resistant protein glutathione transferase (GST)^3^. In an experiment involving *HAT1*, *HAT2* and *HAT3* loss-of-function mutants inoculated with a virus, *HAT1,* *HAT2* and *HAT3* were found to be negative regulators of virus resistance^50^. According to various reports, in Arabidopsis, compared with other proteins, HD-Zip I and II proteins are more sensitive to changes in light quality. When phytochrome senses the light signal reaching the canopy, it mainly participates in the light avoidance reaction by inducing stem growth^51^. In Arabidopsis, shade avoidance is regulated by positive (PIF) and negative (HERL/SISCI) regulators of gene expression to ensure the rapid remodeling of plants to achieve the most suitable architecture for growth in a particular environment^52^. According to the transcriptome sequencing analysis, it was found that the expression of the differentially expressed genes corresponding to heat stress in the transgenic cell line was upregulated, and the genes expressed in response to cadmium ion and other metal ions were also upregulated. At the same time, this line also responded to injury, bacteria, and viruses, indicating that the transgenic cell line has a certain ability to resist external pressure and stresses.

## Supplementary Information

Supplementary Information.

## Data Availability

The datasets generated during and/or analysed during the current study are available from the corresponding author on reasonable request. Data sharing not applicable to this article as no datasets were generated or analysed during the current study. All data generated or analysed during this study are included in this published article [and its Supplementary information files].
