# Supplementary figures and images for: Genetic transformation of LoHDZ2 and analysis of its function to enhance stress resistance in Larix olgensis
Source: Sci Rep. 2022 Jul 27;12:12831. doi: 10.1038/s41598-022-17191-2 (PMC9329289; doi:10.1038/s41598-022-17191-2)

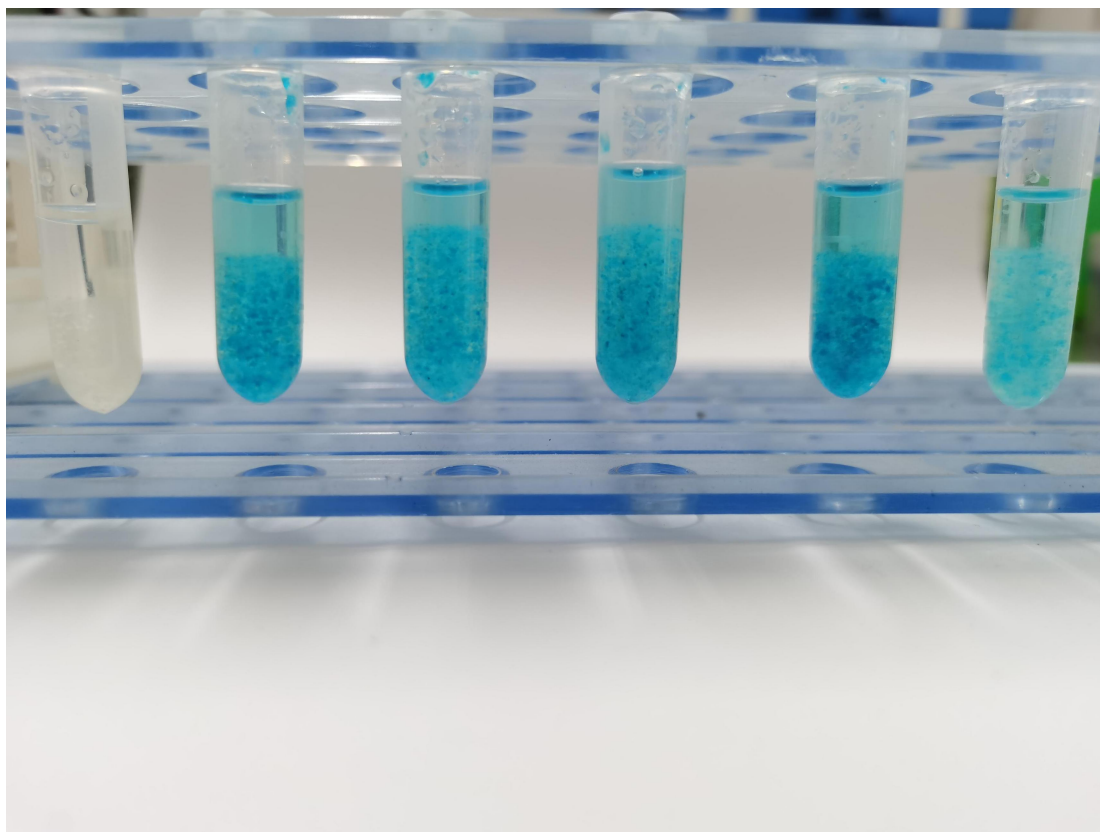

Original image of Figure. 5

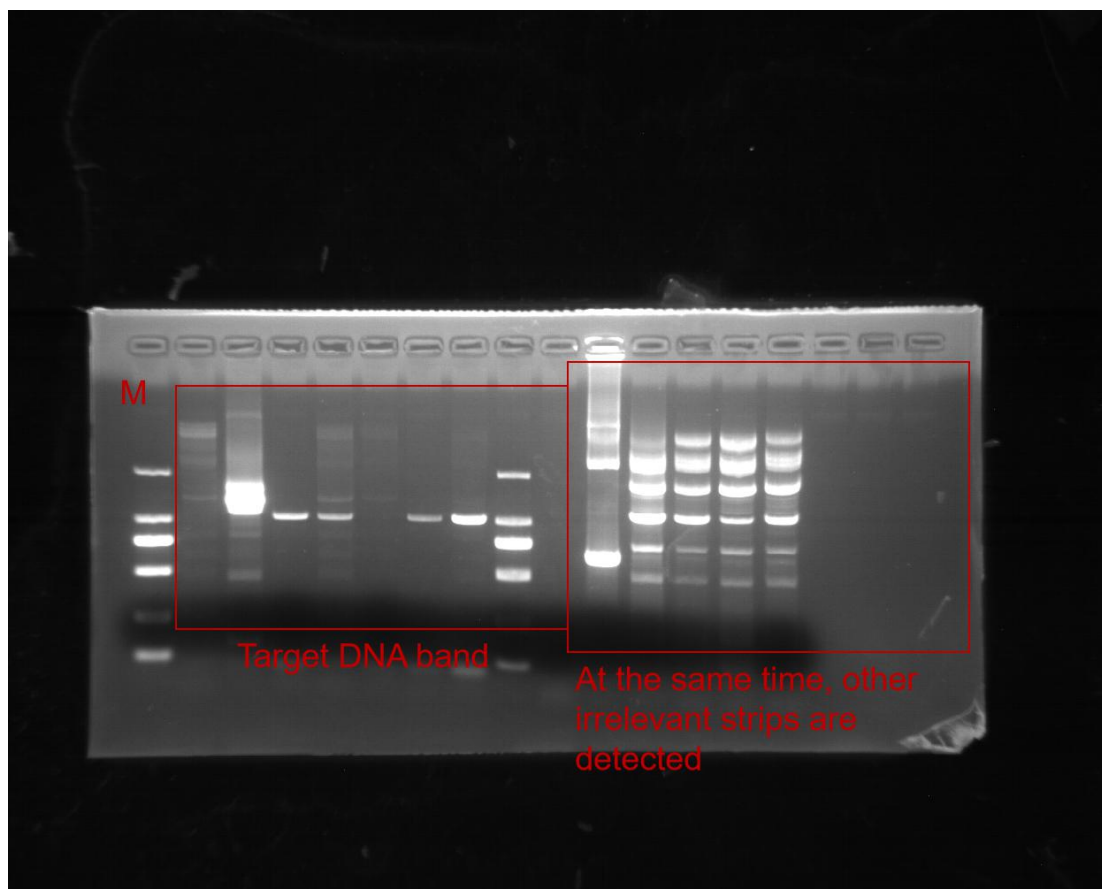

Original image of Figure. 3

Supplement: Supplementary file 1 — Supplementary Information. [file 41598_2022_17191_MOESM1_ESM.pdf]
